# First characterization of methanogens in oral cavity in Malian patients with oral cavity pathologies

**DOI:** 10.1186/s12903-019-0929-8

**Published:** 2019-10-30

**Authors:** Elisabeth Sogodogo, Ogobara Doumbo, Gérard Aboudharam, Bourema Kouriba, Ousseynou Diawara, Hapssa Koita, Souleymane Togora, Michel Drancourt

**Affiliations:** 1 Aix Marseille University, IRD, MEPHI, IHU-Méditerranée Infection, 19-21, Boulevard Jean Moulin, 13005 Marseille, France; 2 IHU Méditerranée Infection, Marseille, France; 3 Department of Epidemiology of Parasitic Diseases, Malaria Research and Training Center, University of Science, Techniques and Technologies of Bamako, Bamako, Mali; 4 Aix-Marseille-University, UFR Odontology, Marseille, France; 5 Centre d’Infectiologie Charles-Mérieux (CICM), Bamako, Mali; 6 National Center of Odonto Stomatology, Faculty of Medicine and Odonto Stomatology, Bamako, Mali

**Keywords:** *Methanobrevibacter oralis*, *Methanobrevibacter smithii*, *Methanobrevibacter massiliense*, Methanogen, Oral cavity, Mali, Africa

## Abstract

**Background:**

The oral cavity of humans is inhabited by several hundreds of bacterial species and other microorganisms such as fungi and archaeal methanogens. Regarding methanogens, data have been obtained from oral cavity samples collected in Europe, America and Asia. There is no study published on the presence of methanogens in the oral cavity in persons living in Africa. The objective of our study was to bring new knowledge on the distribution of oral methanogens in persons living in Mali, Africa.

**Methods:**

A total of 31 patients were included in the study during a 15-day collection period in September. Bacterial investigations consisted in culturing the bacteria in 5% sheep blood–enriched Columbia agar and PolyViteX agar plates. For archaeal research, we used various methods including culture, molecular biology and fluorescent in situ hybridization (FISH).

**Results:**

Eight of 31 (26%) oral samples collected in eight patients consulting for stomatology diseases tested positive in polymerase chain-reaction (PCR)-based assays for methanogens including five cases of *Methanobrevibacter oralis* and one case each of *Methanobrevibacter smithii*, *Methanobrevibacter massiliense* and co-infection *Methanobrevibacter oralis* and *Methanobrevibacter massiliense*.

**Conclusions:**

In this pilot study, we are reporting here the first characterization of methanogens in the oral cavity in eight patients in Mali. These methanogen species have already been documented in oral specimens collected from individuals in Europe, Asia, North America and Brazil.

## Introduction

The oral cavity microbiota is considered to be of heterogeneous origin, including various endo- and exogenous species [1–3]. The oral cavity of humans is inhabited by several hundreds of bacterial species and other microorganisms including unicellular eukaryotes and prokaryotes including fungi and Archaea. Some of these microorganisms have a key role in the development of oral diseases, mainly dental caries and periodontitis [2]. Periodontitis is an inflammatory disease resulting from the polymicrobial infection of the subgingival dental plaque by oral bacteria [4]. Archaea are microorganisms classified among one domain of life, different from the ones including bacteria and eukaryotes [5]. Archaea and more specifically methanogen-producing archaea (herein referred as methanogens) are part of the oral microbiota [6] . Methanogens are archaea with a hydrogenotrophic metabolism requiring the presence of hydrogen (H_2_) to reduce CO_2_ to methane, a process called methanogenesis [7]. Methanogenesis is a unique metabolic process by which carbon dioxide (CO_2_) is reduced to methane (CH_4_) using hydrogen (H_2_) produced by anaerobic bacterial fermentation as an electron donor [7]. Indeed, six species of methanogens belonging to the genera *Methanobrevibacter* have been documented in the oral cavity by polymerase chain-reaction (PCR)-based methods and culture, including *Methanobrevibacter oralis* and *Methanobrevibacter smithii, Methanosphaera stadtmanea*, *Methanosarcina mazeii*, *Methanobacterium curvum/congolense* and *Thermoplasmata* [8, 9]. Previous studies have shown that *M. oralis* is highly dominant with a prevalence greater than 40%, while other methanogens have been detected with a lower prevalence of 10 to 20% [8, 10, 11]. In particular, *M. oralis* has been isolated from the dental plaques of healthy subjects in the oral cavity [12]. One step forward, a recent study disclosed a significant correlation between PCR-detection of methanogens in the oral cavity and tobacco smoking in oral disease-free individuals, illustrating the potential influence of environmental factors of the repertoire of oral cavity methanogens [13]. A molecular study has also shown that methanogens constitute an essential part of the microbiota of the dental root and could participate in the endodontic community in the necrotic root canal [14].

Also, methanogens have been implicated in oral cavity pathologies including *Methanobrevibacter massiliense* and *M. oralis* in cases of periimplantitis [4, 15].

All the studies related to the repertoire of methanogens in the oral cavity have been conducted in specimens collected in individuals in Europe, Asia, North America and Brazil. No study issued from individuals in Africa, living unknown whether the methanogens previously documented in the oral cavity are universally distributed over continents or restricted to certain geographic populations. In order to further study this question, we embarked in studying the presence of methanogens and associated bacteria in oral cavity specimens collected from volunteer patients in Mali, Africa.

## Methods

### Patients and clinical sample collection

This case-series study was reviewed and approved by the Faculty of Medicine, Pharmacy and Odonto-Stomatology Ethics Committee, Bamako, Mali under N°2015/132/CE/FMPOS. This study was conducted in the Stomatology Department of the University Hospital of Bamako, where patients are consulting for oral cavity pathologies with the exclusion of orthodontia. The objective of the study and the sampling protocol were explained to the patients before asking for their consent to participate. A consent document given to each patient and all the patients included in the study, was signed by the patient or one of his parents for children under 18 years. All parental consents were written. Patients who did not sign the contentment form, were excluded from this study. Patients with, gingivitis, periodontitis (either of pulp or peridontal origin) or with dental abscesses who signed the consent form, were included in the study. This is a preliminary and explorative study for a series of pathological clinical cases. The patients were recruited regardless of their recent antibiotics usage status due to its prevalent pre-operative usage in the studied population. This study concerns the presence of methanogens archaea for these cases. Each case was diagnosed by a dentist, before taking samples. Dental X-Ray were made to make the different diagnoses. The classification of periodontal diseases adopted by the dentist was the classification of Armitage et al. [16]. A total of 31 samples of abscesses in 2 cases and dental plaque in 29 cases, were prospectively collected at the University Hospital of Odontology and Stomatology of Bamako in agreed patients who came for odontology consultations. Subgingival plaque samples were collected from each patient by using sterile curettes. The two abscesses were swabbed. These samples were put in a transport medium preserving methanogen viability. This transport medium consisted of (per liter): KCl 0.2 g, CaCl2 0.1 g, MgCl2 0.1 g, KH2PO4 0.2 g, Na2HPO4 1.15 g, NaCl 3 g, ascorbic acid 1 g, uric acid 0.1 g, and glutathione 0.1 g [17]. The pH was adjusted to 7.5 using KOH 10 M and kept at 4 °C. The samples were sent to the IHU Méditerranée Infection for investigating methanogens and bacteria.

### Culture of bacteria

Culture of bacteria was performed at 37 °C in 5% sheep blood-enriched Columbia agar and PolyViteX agar (bioMérieux, Marcy l’Etoile, France) under aerobic and anaerobic atmosphere, for 48 h. All microbial colonies that grew on agar plates were identified by matrix-assisted laser desorption-ionization time-of-flight mass spectrometry (MALDI-TOF-MS) using a Microflex spectrometer (Bruker Daltonics, Bremen, Germany) [18, 19]. The bacteria were tested for their capability in hydrogen production using the following protocol. Briefly, bacteria were cultured in a BD Difco™ Brain heart infusion (Fisher Scientific, USA) [20] at 37 °C in Hungate tubes (Dutscher, Issy-les-Moulineaux, France). All tubes were gassed with nitrogen and incubated at 37 °C for 72 h. Hydrogen was detected by gas chromatography (Perkin Elmer, Toulouse, France) as previously described [21].

### PCR-sequencing-based detection of methanogens

Methanogens were searched by PCR-sequencing. A 0.3-g quantity of acid-washed beads (B106 mm, Sigma, Saint-Quentin Fallavier, France) was added in each tube containing 250 μL of oral cavity sample (abscess or dental plaque), the suspension was shaken to achieve a mechanical lysis in a FastPrep BIO 101 apparatus (Qbiogene, Strasbourg, France) at level 6.5 for 2 min. Then 200 μL of buffer TL and OB Protease Solution (1.5 mL) from the E.Z.N.A. Tissue DNA Kit (OMEGA, bio-tek, Norcross, USA) were added. The mixture was incubated overnight at 56 °C. After a second cycle of mechanical lysis, the mixture was incubated for 60 min at 70 °C. Extracted DNA was eluted with 100 μL of elution buffer and the DNA was stored at − 20 °C. A sterile PBS was used as a negative control for each batch of DNA extraction (a negative control for eight samples). Amplification of the archaeal 16S rRNA gene (primers used: SDArch0333aS15, 5′-TCCAGGCCCTACGGG-3′ and SDArch0958aA19, 5′-YCCGGCGTTGAMTCCAATT-3′) and the methyl-coenzyme M reducer (*mcr*A) gene (primers used: mcrAFor, 5’GCTCTACGACCAGATMTGGCTTGG-3′ and mcrARev, 5′ CCGTAGTACGTGAAGTCATCCAGCA − 3′) genes was performed as previously described [22]. Sequencing reactions (Sangers’ method) were carried-out using the Big- Dye Terminator, version 1.1, cycle sequencing kit DNA according to the manufacturer’s instructions (Applied Biosystems, Foster City, USA). Nucleotide sequences were assembled using Chromas Pro software, version 1.7 (Technelysium Pty Ltd., Tewantin, Australia) and compared to the GenBank database by similarity search using the BLASTN program (http://www.ncbi.nlm.nih.gov/blast/).

### Fluorescent in situ hybridization detection of methanogens

We used a fluorescent in situ hybridization (FISH) protocol derived from a FISH protocol for bacteria. We used PCR-negative samples for control**.** A 10 μL-volume of the sample was deposited on a glass slide, allowed to dry in ambient air then fixed with 20 μL of 4% paraformaldehyde for 30 min. Subsequently, a 20 μL-volume of a 10 g/L solution of lysozyme (Sigma) was deposited on the sample and incubated for 30 min at 37 °C further incubated with 5 μL of proteinase K (Sigma) for 5 min at 37 °C. After washing the slide with distilled water, a 10 μL volume of a solution containing 1 μL of the probe Arch915 labeled with Alexa fluor-546 and specific for Archaeal 16S rRNA gene (10 μmoL/L), 1 μL of the *mcr*A probe (10 μmoL/L), 5 μL of hybridization buffer [4x sodium salt citrate (SSC), 10% of sulfate de dextrane, 1 mM ethylene-diamine-tetraacetic-acid (EDTA), 25% of formamide, 300 ng/mL salmon sperm DNA and 1 x solution de Denhardt] (Sigma-Aldrich), 1 μL of a solution containing 0.1% Tween 20 and 0.1% Triton X-100 (Euromedex, Souffelweyersheim, France) and 2 μL of distilled water. The glass slide was covered with a coverslip glued with Fixogum adhesive (Marabu, Bietigheim-Bissingen, Germany) and incubated at 65 °C for 10 min, then at 37 °C for 20 h. Once the hybridization process was completed, the slide was immersed in a series of SSC baths (4X, 2X, 1X and 0.5X) for 5 min in each bath at room temperature. Finally, the slide was covered with a coverslip and observed at 100X magnification with a fluorescence microscope (Leica DMI 6000, Nanterre, France).

## Results

In this study, oral cavity samples were collected from volunteer patients consulting in a tertiary Odontology Department in Bamako, Mali for chronic periodontitis, gingivitis or cellulitis. A total of 31 patients were included in the study during a 15-day collection period in September 2017. Patients included five children, 10 women and 16 men aged from 4 to 63 years. Pathologies were abscesses of pulp origin in 2 cases and dental plaque in 29 cases (12 gingivitis and 17 periodontitis). We did not include any control patients in this case-series study. The samples consisting in 29 dental plaque and two abscesses, were stored in a transport medium [17].

**Fig. 1:**
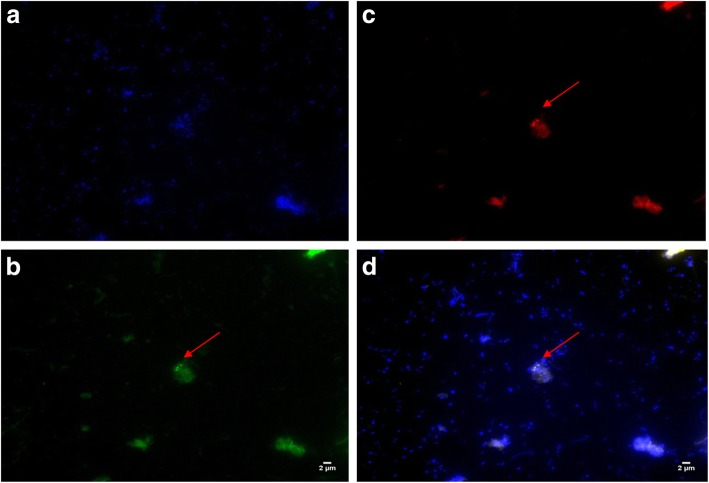
Detection of *Methanobrevibacter oralis* from a chronic periodontitis specimen using FISH. **a** Blue color represents DAPI fluorescence staining any DNA **b** Green color represents *mcrA* probe staining the methanogen *mcrA* gene **c** Red color represents ARC915 fluorescence staining the archaeal DNA **d** Fluorescent *in situ* hybridization combining *mcrA* fluorescence, ARC915 fluorescence and DAPI: the arrow points to archaea. Scale bar, 2 μm

Eight samples (26%) were positive for methanogens by PCR and FISH including five *M. oralis*, one *M. smithii*, one *M. massiliense* and one co-infection with *M. oralis* and *M. massiliense* [12, 22], two methanogens which have been previously documented in cases of periodontitis [23–25] and peri-implantitis [10]. These observations were authenticated by the negativity of the negative controls introduced in the different experiments as well as the fact that concordant observations were made using different observation tools. In particular, bacterial culture allowed the isolation of nine different bacterial species *Delftia acidovorans, Microbacterium oxydans, Pseudomonas putida, Citrobacter freundii, Brevundimonas aurantiaca, Rhizobium radiobacter, Klebsiella pneumoniae, Microbacterium kitamiense* and *Peptoniphilus harei* (Table 1). By using the gas chromatography method, we detected the presence of hydrogen in the culture of two bacteria previously known as hydrogenogen bacteria including *Citrobacter freundii* [26] and *Klebsiella pneumoniae* [27]. However, *Peptoniphilus harei* and *Pseudomonas putida* are not known to produce hydrogen. PCR-sequencing confirmed the presence of *M. oralis* in patients’ n° 2, 3, 4, 6 and 7; of *M. smithii* in patient n° 5 whose clinical data is unavailable [28] and one co-infection *M. oralis* and *M. massiliense* in patient n°1. All these methanogens were identified on the basis of complete sequence identity with references, were detected in association with previously isolated bacteria. The clinical characteristics of the patients and the results are reported in Table 1. These eight PCR-positive samples were all positive by FISH (Fig. 1).

**Table 1:** Detailed clinical information of patients with archaea detected in Odonto stomatology, department, Bamako, Mali. NA: Not Available

Patient	Age range	Locality	Clinical data	Culture	PCR	FISH	Tobacco
1 A 1 B	[60–70]	Badalabougou	Generalized chronic periodontitis	*Delftia acidovorans, Microbacterium oxydans Pseudomonas putida,* *Citrobacter freundii, Brevundimonas aurantiaca, Rhizobium radiobacter*	*Methanobrevibacter oralis* *Methanobrevibacter massilense*	Positive	+
2	[30–40]	Djelibougou	Gingivitis	*Pseudomonas putida, Citrobacter freundii, Microbacterium oxydans,*	*Methanobrevibacter oralis*	Positive	–
3	[60–70]	Darsalam	Moderate generalized chronic periodontitis	*Pseudomonas putida, Citrobacter freundii, Brevundimonas aurantiaca,*	*Methanobrevibacter oralis*	Positive	+
4	[1–10]	Bacodjicoroni	Circumscribed cellulitis of the primary molar 75 (pulpal necrosis)	*Citrobacter freundii, Brevundimonas aurantiaca, Delftia acidovorans, Pseudomonas putida,*	*Methanobrevibacter oralis*	Positive	–
5	[10–20]	Yirimadio	N.A	*Klebsiella pneumoniae, Brevundimonas aurantiaca, Microbacterium oxydans, Pseudomonas putida, Delftia acidovorans,*	*Methanobrevibacter smithii*	Positive	–
6	[60–70]	Daoudabougou	Generalized chronic periodontitis	*Brevundimonas aurantiaca, Delftia acidovorans, Citrobacter freundii, Pseudomonas putida, Rhizobium radiobacter, Microbacterium oxydans, Microbacterium kitamiense*	*Methanobrevibacter oralis*	Positive	–
7	[20–30]	Niarela	Gingivitis	*Rhizobium radiobacter, Pseudomonas putida, Peptoniphilus harei, Delftia acidovorans, Klebsiella pneumoniae,*	*Methanobrevibacter oralis*	Positive	–
8	[40–50]	Korofina nord	Gingivitis	*Pseudomonas putida, Brevundimonas aurantiaca,* *Delftia acidovorans,*	*Methanobrevibacter massilense*	Positive	+

## Discussion

We are reporting the first characterization of methanogens in the oral cavity in patients living in Africa, more precisely in Mali and we are reporting the first case of co-infection by *M. oralis* and *M. massiliense* in the same patient. The observed 20% prevalence of *M. oralis* is lower than the > 40% prevalence previously reported in various studies [8, 9]. This observation most probably relies on the fact that in Mali, there is a systematic prescription of metronidazole before patient care and oral cavity sampling. Indeed, previous studies have shown that methanogens are sensitive to metronidazole and other imidazoles [29]. Previous studies also showed that *M. oralis* was significantly associated with periodontal disease in terms of abundance when comparing patients and controls, as well as diseased and healthy sites in the same patient [23].

The repertoire of three methanogen species here reported in oral cavity specimens collected in patients in Mali, Africa is in agreement with the repertoire recently reported from oral cavity specimens collected in individuals in France, Europa [30]; as well as in North America and Brazil, Asia [4, 8, 14]. Accordingly, two of these methanogens species have also been detected in ancient oral cavity specimens recovered from 100 specimens samples dating from the fourteenth and nineteenth century collected in various archaeological sites in France [31].

Altogether, these data suggest that the oral cavity of populations is hosting an universal core methanogen repertoire comprising of at least *M. oralis*, *M. smithii* and *M. massiliense* which is expected to be recovered from oral cavity specimens collected throughout continents and historical periods; albeit with variations in the relative abundance of the various species in relation with the various environments to which populations are exposed.

## Conclusions

We are reporting here the first characterization of methanogens in the oral cavity in Africa in in particularly in Mali in eight patients. In addition, we are reporting on one case co-infection with *M. oralis* and *M. massiliense* in one patient diagnosed with generalized chronic periodontitis. Further, we observe that *M. oralis*, *M. smithii* and *M. massiliense* are presents in Africa as in other continents, this observation suggests that these three archaea methanogens are frequently represented in situations of periodontitis, whatever the continent.

### Limitations

- This a preliminary, pilot study.
- The fact that only 31 patients were investigated does not allow to draw an actual picture of prevalence and repertoire of oral cavity methanogens in Mali.
- The fact that we were not able to isolate methanogens by culture is one limitation of the present study, as the viability of the three methanogens here detected was not confirmed.
- Based on the proof-of-concept results here reported, further studies designed to assess the viability of methanogens may prospectively include more individuals in additional African countries to overpass these limitations.

## Data Availability

The datasets used and/or analyzed during the current study are available from the corresponding author.

## Abbreviations

EDTA: Ethylene-diamine-tetraacetic-acid
FISH: Fluorescent in situ hybridization
MALDI-TOF-MS: Matrix-assisted laser desorption-ionization time-of-flight mass spectrometry
PCR: Polymerase chain-reaction
SSC: Sodium salt citrate
